# Advances in Integrating Traditional and Omic Biomarkers When Analyzing the Effects of the Mediterranean Diet Intervention in Cardiovascular Prevention

**DOI:** 10.3390/ijms17091469

**Published:** 2016-09-02

**Authors:** Montserrat Fitó, Olle Melander, José Alfredo Martínez, Estefanía Toledo, Christian Carpéné, Dolores Corella

**Affiliations:** 1 Cardiovascular Risk and Nutrition Research (REGICOR Group), Institut Hospital del Mar d’Investigacions Mèdiques (IMIM), 08003 Barcelona, Spain; MFito@IMIM.ES; 2 CIBER de Fisiopatología de la Obesidad y Nutrición (CIBEROBN), 28029 Madrid, Spain; jalfmtz@unav.es (J.A.M.); etoledo@unav.es (E.T.); 3 Department of Clinical Sciences, Lund University, 22100 Lund, Sweden; olle.melander@med.lu.se; 4 Department of Internal Medicine, Skåne University Hospital, 22241 Lund, Sweden; 5 Department of Nutrition and Food Sciences, University of Navarra, 31009 Pamplona, Spain; 6 Department of Preventive Medicine and Public Health, University of Navarra, 31009 Pamplona, Spain; 7 INSERM U1048, Institute of Metabolic and Cardiovascular Diseases (I2MC), Rangueil Hospital, 31442 Toulouse, France; christian.carpene@inserm.fr; 8 Department of Preventive Medicine and Public Health, University of Valencia, 46010 Valencia, Spain

**Keywords:** cardiovascular, biomarkers, omics, Mediterranean diet, gene-diet interactions

## Abstract

Intervention with Mediterranean diet (MedDiet) has provided a high level of evidence in primary prevention of cardiovascular events. Besides enhancing protection from classical risk factors, an improvement has also been described in a number of non-classical ones. Benefits have been reported on biomarkers of oxidation, inflammation, cellular adhesion, adipokine production, and pro-thrombotic state. Although the benefits of the MedDiet have been attributed to its richness in antioxidants, the mechanisms by which it exercises its beneficial effects are not well known. It is thought that the integration of omics including genomics, transcriptomics, epigenomics, and metabolomics, into studies analyzing nutrition and cardiovascular diseases will provide new clues regarding these mechanisms. However, omics integration is still in its infancy. Currently, some single-omics analyses have provided valuable data, mostly in the field of genomics. Thus, several gene-diet interactions in determining both intermediate (plasma lipids, etc.) and final cardiovascular phenotypes (stroke, myocardial infarction, etc.) have been reported. However, few studies have analyzed changes in gene expression and, moreover very few have focused on epigenomic or metabolomic biomarkers related to the MedDiet. Nevertheless, these preliminary results can help to better understand the inter-individual differences in cardiovascular risk and dietary response for further applications in personalized nutrition.

## 1. Introduction

Every day, the concept of “Precision Medicine” or “Personalized Medicine” is becoming more widely known and professionals from various fields are working hard to ensure that the promises that this new vision of medicine, which proposes customizing healthcare, with medical decisions, practices, and/or products tailored to the individual patient, are fulfilled. To do so, omics are essential. Although the concept of precision medicine has been more focused on curing, customizing prevention is also being proposed, given that it is known that, for the health care system, prevention and health promotion is far more cost effective than cure. The concept of “Precision Health” is, therefore, emerging in an attempt to build up a more global vision. Both within Precision Medicine and Precision Health, nutrition plays an essential role and, therefore, recently work has also started on the concept of “Precision Nutrition”, which specifically refers to integrating omics in nutrition so as to achieve a better prevention and/or treatment of disease based on a more customized diet. However, so that Precision Nutrition can become a reality, much more research into integrating the new omics into nutritional epidemiology needs to take place. This will allow us to gather new knowledge that we will gradually be able to apply in this new field. For some diseases, such as cardiovascular diseases (CVD), some important studies have already been undertaken that have allowed us to advance in this knowledge [1,2,3,4,5]. Nevertheless, these reports have to be accompanied by more so as to provide a high level of scientific evidence and to minimize biases. To do so, omics have to be integrated into the dietary controlled randomized trials that are undertaken in order to contribute new knowledge on the influence of diet on the risk of CVD.

These trials are relatively simple to undertake for what are called intermediate phenotypes of cardiovascular risk (plasma lipid concentrations, inflammation markers, blood pressure, etc.). However, for final phenotypes of CVD (CVD incidence, mortality, etc.), these trials are expensive and complex, given that they require a long latency period from when the intervention began to when the so-called hard events (myocardial infarction, stroke, etc.) take place. Fortunately, the results of the PREDIMED study (PREvención con DIeta MEDiterránea)—a randomized, controlled nutritional intervention trial [1]—aimed at assessing the influence of the Mediterranean Diet (MedDiet) on hard cardiovascular events, has helped not only to provide a higher level of evidence on the effects of the MedDiet on cardiovascular prevention, but has also encouraged various research groups throughout the world to begin or plan the design of large nutritional intervention studies focusing on hard cardiovascular events. Those studies that have been designed within the new omics era are now incorporating all these new technologies so as to provide better knowledge. The knowledge that omics can provide to nutritional studies of the MedDiet and CVD is twofold: on the one hand providing more information on the molecular mechanisms through which the MedDiet exercises its beneficial effects and, on the other hand providing information on omic biomarkers (genomic, epigenomic, metabolomic, etc.) for application in Precision Nutrition. In general, in nutritional epidemiology of CVD, three types of biomarkers are used: (1) Biomarkers of dietary exposure; (2) Biomarkers of nutritional status; and (3) Biomarkers of health/disease [6,7]. In this work, we will review present knowledge from randomized trials with the MedDiet, which provide the first scientific level of evidence, as well as the traditional biomarkers of health/disease status and will go deeper into new omics (genomics, transcriptomics, epigenomics, metabolomics) as they promise to revolutionize the identification of new biomarkers (of dietary exposure, of nutrition status, and of health/disease) in nutritional studies into CVD, to present the advances in integrating traditional and omic biomarkers when analyzing the effects of the MedDiet in CVD prevention.

## 2. Advances in Nutrition: Traditional Mediterranean Diet and Cardiovascular Risk

In clinical practice, main efforts are focused on blood pressure and lipid profile check-ups, management of diabetes and obesity, and promotion of smoking cessation. A healthy diet pattern, such as the traditional MedDiet, can be a useful and complementary tool, together with undertaking of physical exercise, to obtain better control of these risk factors. The PREDIMED study was the first primary prevention, randomized, controlled trial to test the long-term effects of the MedDiet on CVD incidence [1]. Participants were randomized into one of three diets: (1) MedDiet supplemented with extra-virgin olive oil (EVOO); (2) MedDiet supplemented with nuts; and (3) control diet (advice on a low-fat diet). The PREDIMED study (www.predimed.es) has provided evidence, for the first time, of the primary prevention for cardiovascular events as a hard composite endpoint (myocardial infarction, stroke, and cardiovascular mortality) [1,2], stroke [1], atrial fibrillation [3], type-2 diabetes [4], and peripheral vascular disease [5], in high cardiovascular risk individuals. We will review present knowledge from randomized trials with the MedDiet, which provide the first scientific level of evidence, on traditional biomarkers of health/disease status and will go deeper into the new omics—genomics, transcriptomics, epigenomics, metabolomics, etc.—as they will be crucial in preventing and treating CVD [7].

## 3. Effects of the MedDiet on Traditional Biomarkers (Classical and Non-Classical Risk Factors)

A decrease in systolic and diastolic blood pressure in both PREDIMED MedDiet interventions was observed after a three-month intervention compared to each baseline [8]. Recently, we have reported that long-term intervention (4.8 years of median follow-up) with a non-energy restricted MedDiet (observed for both groups: one supplemented with EVOO and the other with nuts) did not significantly increase body-weight in comparison with the control low-fat group [9]. Moreover, intervention with MedDiet had a more favorable effect on the slight increase in waist circumference observed as a trend in the three groups [9]. Glucose-related and lipid profile parameters also improved after both three-month MedDiet interventions versus their baselines and versus the low fat diet control group in PREDIMED participants [8,10]. Likewise, lipoprotein subclasses were shifted to a less atherogenic pattern by both MedDiet consumptions versus their baselines, especially the one enriched with nuts [11]. Beneficial effects of the intervention with the MedDiet have also been observed in other populations. Thus, MedDiet intervention, in chronic renal failure patients in Algeria, improved triglycerides, and total cholesterol concentration versus the control group [12] and an enhancement in lipid profile and fibrinogen was observed after the MedDiet versus its baseline [12]. Likewise, in and intervention study carried out in Greece, hypercholesterolaemic an improvement in adherence to the MedDiet in hypercholesterolaemic subjects, was associated with a reduction low-density lipoprotein (LDL) cholesterol, triglycerides, systolic and diastolic blood pressure, coagulation markers, as well as with an increase in high-density lipoprotein (HDL) cholesterol concentration [13]. Accordingly, a meta-analysis of high quality trials with MedDiet, in metabolic syndrome volunteers, showed significant effects of the MedDiet in systolic and diastolic blood pressure, homeostasis model assessment (HOMA), glucose, HDL cholesterol, and triglycerides [14]. Also in Spanish patients with metabolic syndrome, intensive life-style intervention counseling, including MedDiet, resulted in improvements in abdominal circumference, blood pressure, and HDL-cholesterol [15]. Similarly, an updated meta-analysis including 29 intervention trials, found that intervention with MedDiet significantly decreased waist circumference, triglyceride concentrations, fasting glucose, and blood pressure (systolic and diastolic). These beneficial effects were higher in studies of longer duration and carried out in Europe [16]. Although none focused on primary CVD prevention, but on secondary prevention, the CORDIOPREV Study (CORonary Diet Intervention with Olive oil and cardiovascular PREVention), is an ongoing intervention trial with MedDiet versus a low-fat diet on coronary patients from Spain aimed at analyzing the effects of these intervention both on traditional cardiovascular biomarkers and CVD recurrence [17]. Interesting results will be obtained in the next few years. Despite their overall value, some classical cardiovascular risk biomarkers tend to lose predictive power in specific populations such as the elderly [18]. In addition, a percentage of individuals who develop CVD have only one or none of the classical CVD risk factors [19,20] and might even prove to have a worse prognosis [21]. There is, therefore, interest in identifying new biomarkers linked to residual risk for CVD development [22].

Atherosclerosis, which is the major underlying cause of CVD and is associated with many other chronic degenerative diseases, involves several highly interrelated processes, including lipid metabolism, platelet activation, and thrombosis, endothelial dysfunction, inflammation, oxidation, vascular smooth cell activation, matrix metabolism, remodeling, and genetic factors [23]. At present, research into these processes is leading to the emergence of new non-omics and omics biomarkers related to CVD.

LDL cholesterol concentration has long been associated with cardiovascular risk. Moreover, oxidative modifications of the LDL particle may play a key role in atherosclerosis [24] and have been associated with the severity of coronary heart disease [25]. Several follow-up studies have reported that systemic circulating oxidized LDL could be a predictor of acute coronary syndromes in the general population [26,27]. In PREDIMED decreased oxidized LDL was observed three months and one year after both MedDiets versus each baseline [28,29]. In addition, NT-proBNP (a) and Lp (a) plasma concentrations, also presented a one-year reduction compared with participants assigned to a low-fat diet [29]. Although in all the intervention groups of PREDIMED systemic F2-isoprostanes and 8-oxo-deoxiguanosine were reduced after a one-year intervention, in women with metabolic syndrome who improved their diet towards a MedDiet pattern, the decrease in 8-oxo-deoxiguanosine was greater [30]. In addition, both MedDiet interventions increased plasma non-enzymatic antioxidant capacity after a one-year intervention, within the frame of the same study [31]. In a longer three-year follow-up, both MedDiets—particularly the EVOO-rich one—were associated with higher levels of plasma antioxidant capacity [32]. In other studies, it has been reported that adherence to a MedDiet may protect against artery wall production of inflammatory mediators [33]. In this regard, C reactive protein, interleukin-6, soluble vascular cell adhesion molecule 1 (sVCAM1), and soluble intercellular cell adhesion molecule 1 (sICAM1) concentrations decreased after both MedDiets three-month interventions in the PREDIMED study [8]. In addition, T lymphocytes sVCAM1 and C-reactive protein only decreased after the MedDiet supplemented with EVOO, whereas interleukin-6, sVCAM1, and sICAM1, increased in the control group [8]. Similarly, in another study, a two-month hypocaloric MedDiet resulted in reductions in proinflammatory markers, such as the adipose tissue derived retinol-binding protein 4, one of the most important adipokines linked with the metabolic syndrome [34]. Moreover, recent data from the PREDIMED study have demonstrated that has demonstrated the anti-inflammatory effects short-term anti-inflammatory effects of the MedDiet also remain significant after a long-term (more than three years) intervention period [35]. Also it has been reported that, when a habitual diet high in monounsaturated fatty acid, such as the MedDiet, is consumed, a lower post-prandial increase of coagulation factor VII (FVIIc) has been reported in other studies [36,37]. Moreover, some genetic polymorphisms have been linked to this observation [38], showing the interest in the integration of omic data. Although those biomarkers are mainly associated with CVD, some of them may have an important role in other diseases such. Thus a recent meta-analysis has investigated the association between the MedDiet and the main chronic diseases and has found a consistent protection of the MedDiet not only against CVD, but also on cancer [39].

## 4. Omics Integration and New Omic-Based Biomarkers

Despite the progress in the identification of the associations between diet and classical and emerging CVD biomarkers, genetic markers are becoming increasingly important as research advances on inter-individual variability [40,41]. In particular, genetic polymorphisms in relevant genes have been related with each one of the traditional biomarkers previously mentioned. In addition, the underlying mechanisms by which the MedDiet exercises its beneficial effects on CVD are not well known (Table 1) [42,43,44,45,46,47,48]. It is thought that omics integration in the studies analyzing nutrition and CVD will provide new clues regarding these mechanisms [49]. All of this underlines the importance of the joint integration of the several omics and increases interest in undertaking more integrated studies [50]. A deeper integration of omics technologies along with new high-throughput computational methods and a systems biology approach will allow us to identify a better list of biomarkers useful for diagnosis and therapies of cardiovascular diseases and related gene-diet interactions [49,51].

The PREDIMED study provides an ideal framework for obtaining new knowledge on classical and new omic-based biomarkers not only at a single-omic, but also at an integrative multi-omics level. Omic-based biomarkers can be classified as detailed in Table 2 [40,52,53,54,55,56,57,58,59,60].

Omic biomarkers require the obtaining of different types of biological samples for their measurements. The most commonly employed are blood, saliva and urine, although other samples are used such as adipose tissue or other tissues depending on the aims of the study. Although obtaining DNA for genotyping allows general determinations that are independent of the tissue used. Obtaining DNA for epigenetic analyses or RNA for transcriptomic analyses implies an additional difficulty, as methylation and gene expression varies depending on the tissue analyzed. Despite these limitations, it is suitable to obtain and store biological samples in any new nutritional epidemiology studies that are started.

## 5. Omic Biomarkers in the PREDIMED Study and in Other Studies: Gene–MedDiet Interactions

### 5.1. Genomic Biomarkers

Currently, we have increasingly faster and cheaper technology to analyze genetic variability. Thus, we have progressed from studies on candidate genes, in which a few genetic polymorphisms, basically SNPs, were analyzed to genome-wide association studies (GWAs), in which hundreds of thousands of SNPs are analyzed [40]. Moreover, next generation sequencing will provide more detailed information. However, it is still expensive for large samples. Therefore, one of the widely used approaches to combine genetic information provided by different SNPs is the use of the so-called “genetic risk scores” (GRS). There are two types of GRS: unweighted and weighted. The unweighted GRS are built by summing up the number of risk alleles (0 for wild-type, 1 for heterozygous subjects, and 2 for homozygous) genotypes for the selected SNPs. In the weighted approach, the loci are also corrected by the strength of the corresponding association, using the regression coefficients previously obtained for the selected associations.

Genomic biomarkers can be either of intake or of effect or disease risk. Concerning biomarkers of disease, our results in the PREDIMED study, in agreement with other investigations [40,41], have shown an important genetic heterogeneity in determining classical cardiovascular risk factors such as plasma lipid concentrations [61,62,63,64], fasting glucose [62,63,65], inflammatory markers [66], blood pressure [67], and obesity-related measurements [65,68]. Therefore, these results add more evidence to the idea that both traditional and omic biomarkers should be integrated and analyzed together in order to provide better information. In addition, in the PREDIMED study we have found several gene-diet interactions in determining such intermediate cardiovascular risk phenotypes or also called traditional biomarkers [62,63,64]. Although several studies in other populations have analyzed gene-diet interactions focusing on specific foods or macronutrients, very few studies have analyzed gene-diet interactions focusing on the whole MedDiet pattern. Frequently, in observational studies, adherence to MedDiet has is measured using several validated questionnaires [69,70,71] consisting of several questions related to typical foods of the MedDiet. In the PREDIMED study, adherence to MedDiet was measured using a validated 14-item questionnaire [71]. A higher score in this questionnaire indicated greater adherence, 9 points being the mean for the population. In the PREDIMED study, we focused on two SNPs strongly associated with obesity and type-2 diabetes: the FTO-rs9939609 and the MC4R-rs17782313 polymorphisms. We also calculated their additive GRS (unweighted). We found statistically significant gene-diet interactions between the FTO-rs9939609 SNP and the MC4R-rs17782313 SNP, separately, with the MedDiet score in determining type 2 diabetes risk at baseline. Likewise, we found a statistically significant gene-diet interaction between the GRS of these polymorphisms and the adherence to MedDiet in determining type 2 diabetes risk [65]. Thus, when the adherence to MedDiet was low (less than 9 points), these polymorphisms (the minor alleles in a dominant model) were associated with higher risk of type 2 diabetes, both individually and in the GRS. However, when adherence to MedDiet was high, these polymorphisms were not associated with greater type 2 diabetes risk, so reversing genetic susceptibility [65]. Likewise, some other observational studies in different populations have reported gene-MedDiet interactions on body-weight [72,73] or oxidation markers [74].

However, these gene-diet interactions were observed cross-sectionally and they could be subject to bias. The important thing is to discover in the PREDIMED study whether the intervention with the MedDiet is capable of modulating genetic effects in different cardiovascular phenotypes, so providing a higher level of scientific evidence. There are very few dietary intervention trials with MedDiet analyzing gene-diet interactions on cardiovascular phenotypes. We have summarized the current evidence of reported interactions in Table 3 [63,64,75,76,77,78]. Moreover, only the PREDIMED study has analyzed gene-diet interactions from intervention with MedDiet in determining hard cardiovascular end points (total CVD incidence, stroke, myocardial infarction, CVD mortality, etc.).

When we started to carry out these studies, there was no published study in the field of nutrigenetics that had analyzed a gene-diet interaction in determining the incidence of CVD using a randomized and controlled nutrition intervention trial.

In the PREDIMED study, we have found several gene-diet interactions in determining incidence of CVD. Among them, we would like to underline that found with the TCF7L2-rs7903146 C>T SNP and the dietary intervention (MedDiet versus control) in determining stroke incidence [63]. The TCF7L2-rs7903146 polymorphism has been associated with a higher risk of type 2 diabetes, as well as with CVD risk [79,80] in carriers of the variant T-allele. In the PREDIMED trial we showed that MedDiet intervention modulated the effects of the TCF7L2-rs7903146 C>T SNP on stroke incidence. Thus, TT subjects had a higher stroke incidence in the control group (compared with CC individuals), whereas dietary intervention with MedDiet reduced stroke incidence in TT subjects and no differences with CC subjects were detected. This result represents the first report showing that a dietary intervention, in this case the MedDiet, counteracts a genetic risk of stroke.

Moreover, focusing on myocardial infarction, although the results of the PREDIMED study did not show a statistically significant MedDiet protection for myocardial infarction incidence, in a nutrigenetic study in PREDIMED participants [64], we did show the protective effect of the MedDiet in individuals with a certain Max-like protein x (MLX) interacting protein like (*MLXIPL*) variant. In a previous GWAS [81], we found that the minor allele of the *MLXIPL*-rs17145738 SNP was associated with significantly lower triglyceride concentration. This SNP was intergenic and later a functional variant (rs3812316, C771G: Gln241His), in high LD with rs17145738, was described [82]. In PREDIMED, we observed a strong association between the rs3812316-*MLXIPL* and lower triglycerides concentrations in carriers of the minor allele [64]. Moreover, we found a significant gene-diet interaction for this SNP in hypertriglyceridemia. When adherence to MedDiet was high, the protective effect of the minor allele against hypertriglyceridemia was stronger. However, when adherence to MedDiet was low, the protection did not reach statistical significance. More importantly, the protective effect of the minor allele against myocardial infarction was only statistically significant in subjects in the intervention group receiving MedDiet.

In addition to these pioneering studies showing, for the first time, that intervention with MedDiet can counteract the higher risk of stroke associated with the minor allele for the TCF7L2-rs7903146 SNP [63] as well as to enhance the protective effect of the minor allele for the *MLXIPL*-rs3812316 SNP [64] against myocardial infarction, we have published other gene-MedDiet intervention interactions in determining total CVD or stroke [77,78] ,and we are obtaining novel, still unpublished, gene-MedDiet interactions that provide accumulative evidence of relevant dietary modulations on the genetic effects in the CVD risk determination.

### 5.2. Epigenomic Biomarkers

Epigenomics is term is used to describe a diversity of modifications to the genome that do not involve changes in DNA sequence and can result in alteration of gene expression [53]. It constitutes the missing link between genetics, the environment and the outcome phenotypes. The epigenetic marks are reversible, and may allow a quick adaptation to the environment. There are three categories of epigenetic biomarkers based on the main epigenetic regulators: DNA methylation, histone modification, and non-coding RNAs.

Most DNA methylation occurs at cytosine–phosphate–guanine (CpG) dinucleotides, providing marks in the genome by which genes are set to be transcriptionally activated or silenced [83,84] Although some studies have reported dietary modification in DNA-methylation related to diet in different studies [84,85,86,87,88,89], the heterogeneity of the interventions and the small sample size of these preliminary studies requires further replication to validate the findings. Moreover, although in some of these studies [85,89], intervention with MedDiet has been evaluated, taking into account that the main aim of these studies was to test the effect of an energy-restricted MedDiet on weight loss, the statistically significant changes in DNA methylation reported in these studies may be due both to the intervention with MedDiet and to the decrease in weight. Therefore, how the process of DNA methylation-demethylation is regulated by MedDiet remains unclear and more studies are needed to establish epigenomic biomarkers for methylation related to MedDiet intervention. In the PREDIMED study we are currently investigating some of these biomarkers. Similarly, alterations of histone modification (acetylation, etc.) have still to be explored in human intervention studies with MedDiet, and, therefore, new research is needed. In animal models, there is emerging evidence that a high fat diet intervention induces chromatin accessibility changes and that persistent chromatin accessibility changes are associated with histone post-translational modifications [90]. Likewise, the effect of the MedDiet intervention on circulating microRNA (miRNA) and their regulation of gene expression have been largely unexplored. miRNAs have emerged as crucial epigenetic regulators of many process related to CVD [91,92,93,94]. miRNAs are small (18–25 nucleotide) functional non-coding RNAs that regulate gene expression of their target mRNA in a post-transcriptional manner. miRNAs can be detected and measured in different tissues and miRNA expression may be cell specific. However, the discovery that microRNAs circulate in a stable form in plasma, facilitates that circulating microRNAs can serve as new omics biomarkers for CVD [94]. Currently, distinctive patterns of circulating miRNAs have been found for several intermediate and final CVD phenotypes including hypertension, diabetes, stroke, myocardial infarction, etc. [94,95,96]. Numerous groups of researchers are investigating the effect of several dietary interventions on the miRNA circulating profile associated with the CVD phenotypes. However, published results are still scarce and heterogeneous. Among them, it has been reported that subjects with prediabetes consuming a pistachio-supplemented diet in comparison with an isocaloric diet in a randomized crossover nutrition intervention trial, had lower circulating levels of miR-192 and miR-375 compared to the isocaloric diet [97]. Both miRNA have been directly correlated with fasting glucose and insulin. In terms of macronutrients, it has been reported that intervention with a high protein diet modifies the microRNA circulating profile of miR-223, in comparison with a normal protein diet [98]. Likewise, it has been shown that an eight-week trial with a normocaloric diet enriched with PUFAs is associated with changes in the miRNA circulating profile including decreased miR-328, miR-330-3p, miR-221, and miR-125a-5p; and increased miR-192, miR-486-5p, miR-19b, miR-106a, miR-769-5p, miR-130b, and miR-18a [99]. Despite these interesting results supporting the hypothesis that diet can influence the circulating miRNA profile, the influence of a whole dietary intervention with MedDiet on circulating miRNA remains to be evaluated.

Regarding miRNA expression in different tissues, interesting results have been obtained when analyzing miRNA expression in white blood cells (WBC) in metabolic syndrome patients receiving intervention with MedDiet in the RESMENA (The reduction of the metabolyc syndrome in Navarra-Spain) study. In this dietary intervention, the MedDiet was able to induce changes in the expression of let-7b and miR-155-3p (both associated with inflammatory parameters) in WBC [100].

On the other hand, the identification of the respective targets of the circulating or of the tissue-specific miRNAs may provide novel molecular insight. On this regard, SNPs in miRNA target sites have also been demonstrated to have allele-specific effects [101]. Thus, the minor allele of the SNP rs13702 T>C in the lipoprotein lipase (LPL) 3’UTR gene disrupts a miRNA recognition element seed site for the human miRNA-410, resulting in a gain-of-function and lower plasma triglyceride concentrations [101]. Regarding epigenetic biomarkers and omics integration, in the PREDIMED study we have reported a gene-diet interaction involving the mRNA-410 target site polymorphism in the *LPL* gene in determining plasma triglyceride concentrations and stroke incidence [77]. We observed a strong interaction of the rs13702-LPL miRNA target site SNP with monounsaturated fatty acids (MUFA) intake at baseline in determining fasting triglycerides in such a way that the decreasing triglyceride effect of the minor C allele was increased with MUFA intake. Accordingly, after a three-year follow-up period we obtained statistically significant gene-diet interactions with intervention with MedDiet on triglycerides, as expected. Thus, C-carriers had a greater decrease in triglyceride concentrations when allocated to the MedDiet group in comparison with the control group. Moreover, when analyzing the effect of the miRNA-410target site SNP on the incidence of CVD, we observed a significant protection of this SNP only in subjects of the MedDiet. These results are the first of further promising research in this integrative field.

### 5.3. Transcriptomic Biomarkers

There are very few intervention studies analyzing the effect of the whole MedDiet pattern on gene expression. Two intervention studies (including the PREDIMED and another study carried out on subjects with Crohn’s disease) have analyzed gene expression changes in the whole transcriptome using high density arrays [56,102] in response to the intervention with MedDiet, while others have focused on selected candidate genes and intervention with MedDiet or even analyzing the response of typical foods of the MedDiet such as olive oil [103,104,105,106,107]. The results of these studies have been extensively reviewed in recent work [108,109]. Briefly, despite the great diversity among studies, intervention with MedDiet or with EVOO has been related to decreased gene expression of several pro-atherosclerotic genes involved in vascular inflammation, foam cell formation, and oxidative stress [56,100,101,102,103,104,105,106,107]. In the transcriptomic analysis in the PREDIMED study [56] carried out in a subsample after a three-month intervention with MedDiets in comparison with the control group, we detected that the key pathways in the physiopathology of cardiovascular events, such as atherosclerosis, renin-angiotensin, nitric oxide and angiopoietin signaling, were modulated by MedDiet + EVOO, whereas hypoxia and endothelial nitric oxide synthases (eNOS) signaling pathways were modified by both MedDiet; while none of the pathways were modulated by the control group in blood cells. These scarce results are promising, but much work is needed to discover good transcriptomic biomarkers in response to MedDiet.

### 5.4. Metabolomic Biomarkers

The emergence of metabolomics is still recent compared to other omics, but its particular features have contributed greatly to its increasing use [59,110]. Currently, there are a growing number of metabolomic studies in the MedDiet field including the PREDIMED study as well as other studies in other populations [60,101,102,103,104,105,106,107,108,109,110,111,112,113,114,115,116,117,118]. Metabolomics is currently used both to investigate markers of dietary intake and biomarkers of disease [115,116]. Plasma/serum and urine samples of participants are being used depending on the aims and technical issues and more work is needed to standardize the methods and results when comparing different studies on different samples. In the PREDIMED study, we have characterized the dietary walnut fingerprinting in urine samples using an high pressure liquid chromatography coupled with quadrupole time-of-flight mass spectrometry (HPLC-q-ToF-MS) untargeted metabolomics approach [111]. Consumption of walnuts was characterized by 18 metabolites, including markers of fatty acid metabolism, ellagitannin-derived microbial compounds, and intermediate metabolites of the tryptophan/serotonin pathway. Likewise, we characterized the metabolic signature of cocoa consumption in PREDIMED participants [112]. Moreover, we assessed the effect of the MedDiet intervention on the urinary metabolome by comparing a sub-sample of non-diabetic subjects at one and three years of follow-ups [60]. We used multivariate data analysis methods (orthogonal signal correction (OSC) of partial least squares projection to latent structures (PLS) discriminant analysis (DA) (OSC-PLS-DA) and hierarchical clustering analysis (HCA)) to identify the potential biomarker discriminating groups. Our results showed that the most relevant hallmarks related to MedDiet intervention were related to the metabolism of carbohydrates (3-hydroxybutyrate, citrate, and cis-aconitate), creatine, creatinine, amino acids (proline, *N*-acetylglutamine, glycine, branched-chain amino acids, and derived metabolites), lipids (oleic and suberic acids), and microbial cometabolites (phenylacetylglutamine and p-cresol). Likewise, in the RESMENA study, an intervention study with two energy-restricted diets; a diet MedDiet and a control diet (low-fat) carried out on 72 subjects with metabolic syndrome features [113], showed that the MedDiet intervention resulted in significant changes in the plasma metabolic profile at two months (mainly phospholipids and lysophospholipids). However, differences were attenuated at six months.

Overall, our results show that metabolomics may allow the classification of individuals regarding their specific food consumption or dietary patterns. However, more studies are needed to improve the validity and precision of this classification.

On the other hand, we are using metabolomics to identify plasma biomarkers of cardiovascular disease and diabetes in PREDIMED participants. Thus, in a case-cohort study including all the cardiovascular events in the PREDIMED study that occurred after 4.8 years of median follow-up and a random sample of the cohort (10% of participants), we have been able to identify that metabolite profiles characterized by elevated concentrations of plasma acylcarnitines (mainly, short- and medium-chain acylcarnitines) are associated with higher incidence of cardiovascular diseases [117]. Moreover, in this study, we detected that MedDiet intervention modulates the association between baseline plasma acylcarnitines and further cardiovascular risk, mainly for stroke risk, in such a way that MedDiet interventions may diminish the risk of cardiovascular diseases associated with higher plasma concentrations of acylcarnitines before nutritional intervention. Similarly, using the same case-cohort design in the PREDIMED participants, we analyzed the effect of plasma of branched-chain amino acids (leucine, isoleucine, and valine) at baseline and cardiovascular disease risk in the follow-up [118]. We observed a higher incidence of stroke associated with higher branched-chain amino acid concentrations. Moreover, we detected a statistically significant interaction between these concentrations and intervention with MedDiet, in such a way that such intervention counteracted the metabolite effects on stroke. In addition to these metabolomics analyses focused on specific metabolites, a more comprehensive approach analyzing all the metabolites together is needed to better understand the relationships at the whole metabolome level.

### 5.5. Lipidomic Biomarkers

Lipidomics is a subfield of metabolomics that focuses on the overall study of molecular lipids within cells, tissues, and biological fluids. Lipids classification comprises hundreds of thousands distinct lipid molecules which play a role on cellular membranes, signaling molecules, and as energy sources [119]. In a recent revision of the new omics in cardiovascular prevention, lipidomics was included as relevant to evaluate the comprehensive lipid profile beyond the traditional biomarkers for plasma lipid concentrations, and it is increasingly being applied to the CVD field [120]. Although several studies have examined the influence of the diet on lipidomic biomarkers, the number of published studies analyzing the change in lipidomic biomarkers in response to the MedDiet or to its main components, is still very scarce (but the number can be increased if some metabolomics biomarkers are classified as lipidomic biomarkers instead). Among other human studies analyzing lipidomic biomarkers in response to diet, we can mention, as an example for the short-term (postprandial) intervention, the plasma lipidome study carried out to assess the post-prandial effects of dairy fat and soy oil in 16 men [121]. This study showed increased concentrations of plasmalògens (with antioxidant properties), after dairy but not soy meals. With regard mid-term assays, in a two-month trial with fatty fish or lean fish in individuals who had suffered a myocardial infarction or unstable ischemic attack, a lipidomic study was performed [122]. On one hand, species including including ceramides, lysophosphatidylcholines, glycerophospholipids, phosphatidylcholines, and lysophosphatidylethanolamines were decreased in the group on fatty fish diet, and on the other hand in the lean fish intervention cholesterol esters and specific long-chain triglycerides increased. In another study [123], the effects of n-3 fatty acid and polyphenol rich two-month diets on plasma and HDL particle lipidomic patterns, in high cardiovascular risk volunteers, were studied. Associations among clinical variables and lipid molecular species were described, mainly after the diets high in n-3 fatty acids and polyphenols.

Interestingly, a recent study has reported the effect of intervention with a whole diet (Nordic diet) versus a control diet on the fasting plasma lipidomic profile in subjects with metabolic syndrome [124]. Statistically significant changes in 21 plasma lipids were observed between the intervention groups at 12 weeks, including increases in plasmalogens and decreases in ceramides in the healthy Nordic diet group. However, at the end of the study, changes in lipidomic profiles did not differ between the groups. Analyzing the factors contributing to this, including small sample size or other genetic and environmental factors will help to better understand the results.

### 5.6. Multi-Omics Integration in the Response to MedDiet and Cardiovascular Risk

Currently, there is a great interest in omics integration in nutritional epidemiology and cardiovascular prevention [125,126]. Some recent studies in animal models [126] and plants [127] have provided examples of integrated multi-omic analyses, and there are some observational human studies integrating two [128,129,130,131] or three omics (genomics, epigenomics, and metabolomics) [132]. However, multi-omic integration in large nutritional intervention studies is still extremely scarce. In addition to the high cost limitation of obtaining omics data from large sample sizes, there are also important computational limitations to integrating multidimensional omic data coming from GWAs, epigenome-wide methylation studies, genome-wide-transcriptomics, metabolomics, proteomics, and even metagenomics [133]. However, important advances are coming [134,135] and the PREDIMED study provides an ideal framework for multi-omics analyses and for obtaining new knowledge on Nutritional Genomics and its association with incidence of cardiovascular diseases as well as for better understanding the contribution of intermediate cardiovascular biomarkers. Currently, we and other groups are working on such integration in the field of the responses to the MedDiet intervention in determining cardiovascular biomarkers and disease risk to provide novel and interesting findings.

## 6. Conclusions

In summary, the protective effect of the MedDiet on CVDcan be explained by the beneficial effect of this diet on classical risk cardiovascular factors and non-classical ones. Omics integration, both at a single-omic level or better still at a multi-omics level, will be crucial to better understand the mechanisms behind the protective effects of the MedDiet as well as the inter-individual differences in CVD risk and dietary response for further applications in nutritional epidemiology and in personalized nutrition or precision nutrition.

## Figures and Tables

**Table 1:** Underlying mechanisms of the beneficial effects of the Mediterranean diet on cardiovascular diseases (CVD).

Underlying Mechanisms	References
1. Richness in antioxidants	
1.1. Protects blood and tissue components from oxidative stress	[42]
1.2. Limits the oxidation of unsaturated fatty acids during intestinal transit	[42]
2. MUFA ^1^ and PUFA ^2^ content in membranes preserves membrane fluidity and functionality	[43]
3. Richness in nitrates	
3.1. Production of nitrolipids by the nitration of unsaturated fatty acids	[44]
3.2. Nitric oxide generation from the nitrate-nitrite-nitric oxide pathway	[37]
4. Modulation of microbial populations and activities	[45,46]
5. Temporal distribution of food consumption throughout the day	[47]
6. Synergistic interactions and cumulative effects betwee different foods and nutrients	[42]
7. Modulation of gene expression	[48]
8. Modulation of metabolite production	[48]

^1^ MUFA, monounsaturated fatty acids; ^2^ PUFA, polyunsaturated fatty acids.

**Table 2:** Classification of new omic-based biomarkers.

Omic-Based Biomarkers	Description	References
*Genetic biomarkers*	Based on changes in DNA, single nucleotide polymorphisms (SNP). Examples:	
	SNPs in the lactase gene (LCT) as proxies of milk consumption in Mendelian randomization analyses.	[52]
	SNPs in the lipoprotein lipase (LPL) gene as biomarkers of genetic risk of stroke.	[40]
*Epigenetic biomarkers*	Biomarkers based on the main epigenetic regulators: DNA methylation, histone modification, and non-coding RNAs. Examples:	
	DNA hypermetylation or hypomethylation of specific genes depending on food intake; Levels of circulating microRNAs associated with several nutrition-related diseases.	[53,54]
*Transcriptomic biomarkers*	Biomarkers based on RNA expression (whole transcriptome or differences in the expression of selected genes). Example:	[55]
	Differences in the gene expression profile in subjects following a Mediterranean diet in comparison with control subjects.	[56]
*Proteomic biomarkers*	Biomarkers based on the study of the proteome.	[57]
*Lipidomic biomarkers*	Biomarkers based on the study of the lipidome (comprehensive analysis of the molecular lipid species).	[58]
*Metabolomic biomarkers*	Biomarkers based on the study of the metabolome [the entire small molecule (metabolite) component of a system]. Metabolites (including peptides, lipids, nucleotides, carbohydrates, amino acids, and many other classes of small molecules) are generally defined as having an atomic mass of less than 1.5 kDa and can be exogenous, endogenous, or derived from the microbiome. Example:	[59]
	The ^1^H NMR urinary profile in subjects following a traditional Mediterranean diet in comparison with the urinary profile of subject on a low fat diet.	[60]

**Table 3:** Intervention studies with Mediterranean diet (MedDiet) and modification of the intervention effect by genetic variants on cardiovascular risk factors and disease.

Reference	Population Analyzed	Phenotype Analyzed	Study Characteristics	Main Results
Corella et al., 2013 [63]	7018 high cardiovascular risk subjects participating in the PREDIMED study	Stroke incidence	Randomized controlled trial with MedDiet (two groups pooled) versus a control diet (4.8 years of median follow-up)	The association between the TCF7L2-rs7903146 (C>T) polymorphism and stroke was modulated by the intervention with MedDiet. TT subjects had a higher stroke incidence in the control group (*p* = 0.006 compared with CC), whereas dietary intervention with MedDiet reduced stroke incidence in TT homozygotes (*p* = 0.892 compared with CC).
Ortega Azorín et al., 2014 [64]	7166 high cardiovascular risk subjects participating in the PREDIMED study	Myocardial infarction incidence	Randomized controlled trial with MedDiet (two groups pooled) versus a control diet (4.8 years of median follow-up)	The association between the rs3812316 C>G SNP and myocardial infarction incidence was modulated by the intervention with MedDiet. Carriers of the G allele had significantly lower incidence of myocardial infarction only in the MedDiet intervention group.
Gómez-Delgado et al., 2014 [75]	507 metabolic syndrome (MetS) patients selected from the CORDIOPREV clinical trial	Triglycerides and high sensitivity C-reactive protein (hsCRP)	Randomized trial: MedDiet, compared with a low-fat diet (1 year of follow-up)	The rs1800629 polymorphism at the TNFA gene interacted with intervention with MedDiet to influence triglyceride metabolism and inflammation status in MetS subjects. The decrease in triglycerides and hsCRP was statistically significant in G/G subjects compared with carriers of the minor A-allele.
Di Daniele et al., 2014 [76]	40 male patients with chronic kidney disease	Homocysteine levels and other biochemical parameters	Dietary intervention with an Italian Mediterranean organic diet (IMOD) versus low-protein diet (LPD) for 14 days	They found a significant interaction between MTHFR C667T polymorphism and the IMOD on homocysteine levels compared to LPD The IMOD resulted in significant improvement of homocysteine levels in TT.
Corella et al., 2014 [77]	7187 high cardiovascular risk subjects participating in the PREDIMED study	Fasting triglycerides and stroke incidence	Randomized controlled trial with MedDiet (two groups pooled) versus a control diet (4.8 years of median follow-up)	The rs13702 T>C in the 3′ untranslated region of the LPL gene interacted with the intervention with MedDiet in determining changes in triglycerides and stroke incidence. The decreasing effect of the C allele on triglycerides and stroke incidence was only significant in the MedDiet intervention group.
Corella et al., 2016 [78]	3671 non-diabetic subjects participating in the PREDIMED study	Type-2 diabetes incidence	Randomized controlled trial with MedDiet (two groups pooled) versus a control diet (4.8 years of median follow-up)	The CLOCK-rs4580704 C>G SNP was associated with incidence of type-2 diabetes, with variant allele (G) carriers showing decreased incidence (dominant model) compared with CC homozygotes. However, this protection was more significant in the MedDiet intervention group (*p* < 0.001) than in the control group (*p* = 0.818).

PREDIMED: PREvención con DIeta MEDiterránea; CORDIOPREV: CORonary Diet Intervention with Olive oil and cardiovascular PREVention; TNFA: human tumor necrosis factor α.
